# The *UCP2 -866G/A, Ala55Val and UCP3 -55C/T* polymorphisms are associated with premature coronary artery disease and cardiovascular risk factors in Mexican population

**DOI:** 10.1590/1678-4685-GMB-2017-0008

**Published:** 2018-05-21

**Authors:** Ricardo Gamboa, Claudia Huesca-Gómez, Vanessa López-Pérez, Rosalinda Posadas-Sánchez, Guillermo Cardoso-Saldaña, Aida Medina-Urrutia, Juan Gabriel Juárez-Rojas, María Elena Soto, Carlos Posadas-Romero, Gilberto Vargas-Alarcón

**Affiliations:** 1 Instituto Nacional de Cardiología Instituto Nacional de Cardiología Department of Physiology MexicoD.F. Mexico Department of Physiology, Instituto Nacional de Cardiología “Ignacio Chávez”, Mexico D.F., Mexico; 2 Instituto Nacional de Cardiología Instituto Nacional de Cardiología Department of Endocrinology MexicoD.F. Mexico Department of Endocrinology, Instituto Nacional de Cardiología “Ignacio Chávez”, Mexico D.F., Mexico; 3 Instituto Nacional de Cardiología Instituto Nacional de Cardiología Department of Molecular Biology MexicoD.F. Mexico Department of Molecular Biology, Instituto Nacional de Cardiología “Ignacio Chávez”, Mexico D.F., Mexico; 4 Instituto Nacional de Cardiología Instituto Nacional de Cardiología Department of Immunology MexicoD.F. Mexico Department of Immunology, Instituto Nacional de Cardiología “Ignacio Chávez”, Mexico D.F., Mexico

**Keywords:** *UCPs* polymorphisms, premature coronary artery, cardiovascular risk, Mexican population

## Abstract

We examined the role of *UCP* gene polymorphisms as susceptibility markers for premature coronary artery disease (pCAD). The *UCP2 Ala55Val* (*C/T* rs660339), *UCP2* -866G/A (rs659366), and *UCP3* -*55C/T* (rs1800849) polymorphisms were genotyped in 948 patients with pCAD, and 763 controls. The distribution of the *UCP2 A55V* (*C/T* rs660339) and *UCP3 -55* (rs1800849) was similar in patients and controls. However, under a recessive model, the *UCP2 -866* (rs659366) *A* allele was associated with increased risk of developing pCAD (OR = 1.43, *Pc* = 0.003). On the other hand, patients with pCAD and *UCP2 A55V* (rs660339) *TT* showed high levels of visceral abdominal fat (VAF) (*Pc* = 0.002), low levels of subcutaneous abdominal fat (SAF) (*Pc* = 0.001) and high VAT/SAT ratio (*Pc* < 0.001). Also, patients with *UCP2 -866* (rs659366) *AA* showed increased levels of VAF (*Pc* = 0.003), low levels of SAF (*Pc* = 0.001) and a high VAT/SAT ratio (*Pc* = 0.002), whereas patients with the *UCP3 -55* (rs1800849) *TT* presented high levels of VAF (*Pc* = 0.002). The results suggest the association of the *UCP2 -866* (rs659366) polymorphism with risk of developing pCAD. Some polymorphisms were associated with abdominal fat levels and cardiovascular risk factors.

## Introduction

The incidence and prevalence of coronary artery disease (CAD) has been increasing in recent decades, becoming one of the leading causes of mortality worldwide. It is known that obesity is one of the main factors associated with CAD (Makedou *et al.*, 2009; Pischon *et al.*, 2009; Center for Disease Control and Prevention, 2010). Adipose tissue, in particular the intra-abdominal visceral fat, is able to synthesize and release a variety of hormones and cytokines, which are active molecules relevant in the development of CAD. Furthermore, excess adipose tissue is associated with factors that increase the risk of developing CAD such as hyperinsulinemia, insulin resistance, hypertension, and dyslipidemia (Kil *et al.*, 2005; Muzzio *et al.*, 2005). There is evidence suggesting that obesity is associated with cardiac hemodynamics. So, obesity is linked to hyperdynamic circulation, which maintains the metabolic demand caused by excessive fat deposition. Several studies have shown a positive association between body weight and CAD (Vasan, 2003; Zamboni *et al.*, 2005). Moreover, energy expenditure is a complex trait comprised of the resting metabolic rate, the energy expenditure due to physical activity and diet-induced adaptive thermogenesis. In this context, uncoupling proteins (UCPs) are a family of mitochondrial transporters, which play a crucial role in the process of adaptive thermogenesis (Gronek and Holdys, 2013). UCP function is primarily the uncoupling of the mitochondrial oxidative phosphorylation by promoting the leakage of protons across the inner mitochondrial membrane without their passing through the charge path to synthesize ATP (a process involved in the production of heat). As a result of this decoupling mechanism, a mobilization of stored triglycerides ensues, which plays an important role in fat metabolism.

UCPs are integral proteins located in the mitochondrial inner membrane; they have a molecular mass ranging from 31 kDa to 34 kDa (Ledesma *et al.*, 2002). The uncoupling protein UCP-2 is widely expressed in various human tissues, such as the spleen, thymus, leukocytes, macrophages, bone marrow and stomach (Boss *et al.*, 1997; Fleury *et al.*, 1997). In this regard, *UCP-2* mRNA levels in these tissues and in intra-abdominal subcutaneous adipose tissue are lower in obese subjects compared to lean subjects. On the other hand, several studies have already described that UCP-2 has an anti-atherogenic effect in the vascular wall (Blanc *et al.*, 2003), it improves tolerance to cardiac ischemia (McLeod *et al.*, 2005; Cheurfa *et al.*, 2008) and protects cardiomyocytes from oxidative stress induced cell death (Teshima *et al.*, 2003). In contrast, the uncoupling protein UCP-3 is highly specific of the skeletal muscle, and it has been suggested that it is one of the main regulators of adaptive thermogenesis in humans (Argiles *et al.*, 2002; Schrauwen *et al.*, 2002). Some studies have reported associations between uncoupling protein *UCP’s* polymorphisms and type 2 diabetes mellitus (T2DM). Particular attention has been focused on the *UCP-1 -3826A/G* (rs1800592), *UCP2 -866G/A* (rs659366), *UCP2 Ala55Val* (*C/T*; rs660339) and *UCP3 -55C/T* (rs1800849) polymorphisms. However, the results of these studies are controversial with positive and negative associations (Jia *et al.*, 2009, Jia *et al.*, 2010; De Souza *et al.*, 2011; Brondani *et al.*, 2012). This point is of great relevance since it has been observed that almost 70% of patients with diabetes or impaired glucose tolerance present cardiovascular disease (Norhammar *et al.*, 2002). Also, some studies have shown that polymorphisms of the *UCP* genes may contribute to metabolic disorders with major effects on energy metabolism. The polymorphism *-866 G/A* (rs659366) has been significantly associated with asymptomatic carotid artery atherosclerosis in women (Oberkofler *et al.*, 2005) and with CAD in men (Dhamrait *et al.*, 2004). It is known that acute coronary syndromes are the result of a progressive transformation of fatty streaks in atherosclerotic plaques. In these patients, surgery has proven to be a treatment option, which improves symptoms, quality of life and prognosis. In this context, it is important to study the role of UCPs, given that these uncoupling proteins are actively involved in the storage of fat, which constitutes a major cardiovascular risk factor. Therefore, the identification of variants in the *UCP* genes (*UCP2 -866, UCP2 A55V* and *UCP3 -55C/T*) in patients with CAD will aid in evaluating their participation in the predisposition to this disease.

## Subjects and Methods

### Subjects and measures

All participants gave written informed consent. The study complied with the Declaration of Helsinki and was approved by the Ethics Committee of the Instituto Nacional de Cardiología Ignacio Chávez (INCICh). The primary aim of the Genetics of Atherosclerotic Disease (GEA) Study is to investigate genetic factors associated with pCAD, subclinical atherosclerosis (SA) and other coronary risk factors in the Mexican population. All GEA participants are unrelated and of self-reported Mexican-mestizo ancestry (for at least three generations). Incidentally, a Mexican-mestizo is defined as someone born in Mexico who is a descendant of both the original autochthonous inhabitants and Caucasian (predominantly Spaniards) and/or African individuals. We analyzed 1711 individuals, 948 diagnosed with pCAD and 763 healthy unrelated controls (with negative calcium score by computed tomography). pCAD was defined by personal history of myocardial infarction, angioplasty, bypass surgery, or coronary stenosis > 50% (determined by angiography). Selection was performed among the out-patients and patients attending for diagnostic or therapeutic catheterization. Only patients with pCAD (age at diagnosis < 55 years in men and < 65 in women) who did not experience acute cardiovascular events in the three months prior to the study were included. Also, patients with congestive heart failure, liver disease, kidney cancer, untreated dysthyroidism and those with corticosteroid treatment were not included. Controls were apparently healthy asymptomatic individuals without family history of pCAD; they were recruited from blood bank donors and through brochures posted at social service centers. Exclusion criteria for controls included congestive heart failure, liver, renal, thyroid or oncological disease. The selection of the patients and controls for the GEA study was described in a previous study (Villarreal-Molina *et al.*, 2012). All participants filled out standardized questionnaires to provide demographic information, level of education, income, family and personal history of cardiovascular disease, dietary habits, physical activity, alcohol consumption and use of drugs and supplements.

Blood samples were obtained from subjects after a 12-h fasting period. Next, they were centrifuged to separate serum samples. Serum biochemical profiles [total cholesterol, triglycerides, high-density lipoprotein-Cholesterol (HDL-C), apolipoprotein A (Apo A), apolipoprotein B (Apo B) and glucose] were measured with standardized enzymatic procedures using a Hitachi 902 auto-analyzer (Hitachi Ltd., Tokyo, Japan). LDL-C was estimated using the De Long *et al.* (1986) formula. Accuracy and precision of lipid measurements in our laboratory are under periodic surveillance by the Centers for Disease Control and Prevention service (Atlanta, GA, USA). Uric acid, insulin, alanine aminotransferase (ALT), aspartate aminotransferase (AST), alkaline phosphatase (ALP), and creatinine were measured as well. Plasma insulin concentrations were determined by a RIA (Millipore; RIA Kit, Cat. No. HI-14K, MO, USA), the intra- and interassay CV values were 2.1 and 6.8%, respectively. The homeostasis assessment model of insulin resistance (HOMA-IR) was calculated using the formula: [fasting insulin (pmol/L) x times / fasting glucose (mmol/L)]/ 135. Samples for each human subject were analyzed as a single batch. Specifically, the interassay coefficient of variation for the assays was less than 5%.

### Computed tomography of the chest and abdomen

Computed tomography of the chest and abdomen were performed using a 64-channel multi-detector helical computed tomography system (Somatom Sensation, Siemens) and interpreted by experienced radiologists. Scans were read to assess and quantify three parameters: 1) Coronary artery calcification (CAC) score using the Agatston method (Mautner *et al.*, 1994); 2) total abdominal fat (TAF), subcutaneous abdominal fat (SAF) and visceral abdominal fat (VAF) as described by Kvist *et al.* (1988), in order to estimate visceral to subcutaneous adipose tissue ratio (VAT/SAT); and 3) hepatic to splenic attenuation ratio (LSAR) as described by Longo *et al.* (1993). All patients and a group of 950 healthy controls underwent a tomography examination. Remarkably, 187 subjects in the apparently healthy control group presented positive CAC (CAC score > 0) and were considered as individuals with subclinical atherosclerosis. These subjects were not considered in the analysis. The control group only included individuals with negative CAC (n = 763).

### DNA preparation

Genomic DNA was extracted from whole blood containing EDTA by standard techniques. The *UCP2 Ala55Val* (*C/T* rs660339), *UCP2* -866G/A (rs659366), and *UCP3* -*55C/T* (rs1800849) polymorphisms were genotyped using 5’ exonuclease TaqMan genotyping assays on an ABI Prism 7900HT Fast Real-Time PCR system, according to manufacturer’s instructions (Applied Biosystems, Foster City, CA, USA)

### Statistical analysis

All calculations were performed using SPSS version 18.0 (SPSS, Chicago, Il, USA) statistical package. Means ± SD and frequencies of baseline characteristics were estimated. Chi-square test was used to compare frequencies and ANOVA and Student’s *t*-test was used to compare means. Variables with skewed distribution were shown as median (minimum-maximum) and analyzed using a non-parametric test. Logistic regression analysis was used to test for polymorphism associations with pCAD under inheritance models, adjusted by age, gender, body mass index (BMI), and HDL-C levels. ANCOVA was used to determine associations between the polymorphisms and some clinical and metabolic variables, adjusting for the significant confounding factors like age, gender, smoking habit and physical activity as appropriate. Bonferroni correction was employed to control for multiple testing. The *p*-values for associations of the polymorphisms with pCAD were corrected by three comparisons, for associations with subcutaneous, visceral and total fat were corrected by four comparisons and for associations with cardiovascular risk factors were corrected by seven comparisons. The corrected p values are indicated as *Pc*. Genotype frequencies did not show deviation from Hardy-Weinberg equilibrium (HWE) (*P* > 0.05). Pairwise linkage disequilibrium (LD, D’) estimations between polymorphisms and haplotype reconstruction were performed with Haploview version 4:1 (Broad Institute of Massachusetts Institute of Technology and Harvard University, Cambridge, MA, USA).

## Results

General clinical characteristics of the population are shown in Tables 1 and 2. The statistical differences between groups are shown.

**Table 1:** Demographic and clinical characteristics of the study population

	Control group N= 763	pCAD group N= 948	*P*
Sex W/M (%)	80.1/19.9	19.1/80.9	< 0.001
Age (years)	52.2 ± 8.9	53.5 ± 7.8	0.002
Body Mass Index (kg/cm2)	28.1 ± 4.6	28.5 ± 4.1	0.245
Systolic Pressure (mmHg)	114.5 ± 16.6	118.2 ± 18.2	< 0.001
Diastolic Pressure (mmHg)	70.7 ± 8.9	72.4 ± 9.8	0.001
Overweight (%)	45.6	48.0	< 0.001
Obesity (%)	29.5	33.9	< 0.001
Smoker (%)	48.4	49.5	< 0.001
HT (%)	17.3	66.4	< 0.001
Hepatic steatosis (%)	29.7	26.8	0.289
VAF/SAF (cm^2^)	0.554 (014-3.21)	0.721 (0.14-4.52)	< 0.001
SAF (cm^2^)	304.94 (64-713)	260.22 (57-774)	< 0.001
VAF (cm^2^)	147.89 (27-473)	172.60 (23-504)	< 0.001
Total Abdominal Fat (cm^2^)	452.90 (91-1032)	432.44 (113-1031)	0.071

Data are expressed as means ± SD, percentage or median (min-max).

HT: Hypertension, SAF: Subcutaneous abdominal fat, VAF: Visceral abdominal fat.

**Table 2:** Metabolic characteristics of the study population

	Control group N= 763	pCAD group N= 948	*P*
Total-Cholesterol (mg/dL)	192.09 ± 35.8	165.83 ± 46.92	< 0.001
HDL-C (mg/dL)	50.06 ± 14.39	41.28 ± 10.85	< 0.001
LDL-C (mg/dL)	116.46 ± 31.22	96.31 ± 38.92	< 0.001
TG (mg/dL)	160.78 ± 100.34	175.83 ± 101.32	0.007
Glucose (mg/dL)	96.08 ± 31.20	103.06 ± 34.74	< 0.001
Insulin (mg/dL)	18.89 ± 9.63	23.06 ± 14.97	< 0.001
HOMA-IR	4.54 ± 2.95	5.82 ± 4.68	< 0.001
Uric acid (mg/dL)	5.10 ± 1.31	6.40 ± 1.61	< 0.001
Creatinine (mg/dL)	0.78 ± 0.17	1.00 ± 0.17	< 0.001
ALT (IU/L)	27.36 ± 19.55	29.8 ± 19.97	0.023
AST (IU/L)	27.35 ± 12.62	28.07 ± 10.59	0.606
ALP (IU/L)	84.59 ± 25.95	80.49 ± 25.49	0.005
ApoB (mg/dL)	92.01 ± 27.00	83.10 ± 31.05	< 0.001
ApoA (mg/dL)	143.03 ± 34.39	127.34 ± 27.00	< 0.001
T2DM (%)	9.7	23.1	< 0.001

Data are expressed as means ± SD. HDL-C: High density lipoprotein-Cholesterol, LDL-C: Low density lipoprotein-Cholesterol, TG: Triglycerides, HOMA: Homeostatic model in insulin resistance, ALT: Alanine aminotransferase, AST: Aspartate aminotransferase, ALP: Alkaline phosphatase, T2DM: Type 2 Diabetes mellitus

Observed and expected frequencies in the studied polymorphisms were in HWE. The distribution of the *UCP2 A55V* (rs660339) and *UCP3 -55* (rs1800849) polymorphisms was similar in patients with pCAD and healthy controls in all the models analyzed (Table 3). However, under the recessive model adjusted for age, gender, BMI, and HDL-C, the *UCP2 -866* (rs659366) polymorphism was associated with an increased risk of developing pCAD (OR = 1.43, 95% CI:1.15-1.78, *Pc* = 0.003). Statistical power to detect associations with *UCP2 Ala55Val* (*C/T* rs660339), *UCP3* -*55C/T* (rs1800849), and *UCP2* -866G/A (rs659366) was 0.20, 0.06 and > 0.80, respectively, as estimated with QUANTO software (biostats.usc.edu/Quanto.html).

**Table 3:** Association of *UCP* polymorphisms with pCAD.

	N (%)			MAF	Model	OR (95% CI)	*Pc*
*UCP2 A55V*	*C/C*	*C/T*	*T/T*				
Control	191 (25.0)	394 (51.7)	178 (23.3)	0.491	Dominant	0.095 (0.76-1.19)	0.682
(N= 763)							
pCAD	256 (27.1)	459 (48.7)	228 (24.2)	0.485	Recessive	1.15 (0.92-1.43)	0.193
(N= 943)					Additive	1.09 (0.88-1.35)	0.114
*UCP2 -866*	*G/G*	*G/A*	*A/A*				
Control	222 (29.1)	377 (49.4)	164 (21.5)	0.461	Dominant	0.82 (0.65-1.03)	0.098
(N= 763)							
pCAD	211 (22.2)	501 (52.8)	236 (24.9)	0.513	Recessive	1.43 (1.15-1.78)	**0.003**
(N= 943)					Additive	0.68 (0.53-0.85)	**< 0.001**
*UCP3 -55*	*C/C*	*C/T*	*T/T*				
Control	552 (72.3)	192 (25.2)	19 (2.5)	0.150	Dominant	1.29 (0.67-2.49)	0.432
(N= 763)							
pCAD	708 (74.7)	219 (23.3)	18 (1.9)	0.134	Recessive	1.14 (0.91-1.41)	0.229
(N= 943)					Additive	1.15 (0.93-1.42)	0.362

pCAD: premature coronary artery disease. MAF: Minor Allele Frequency. The models were adjusted by age, gender, BMI, and HDL-C.

Bonferroni correction was made multiplying by 3 comparisons (*Pc*). Significant p values are in bold.

The association of the polymorphisms with TAF, SAF and VAF was analyzed in pCAD patients (Table 4). In this analysis, patients with the *UCP2 A55V* (rs660339) *TT* genotype presented high levels of VAF (*Pc* = 0.002), low levels of SAF (*Pc* = 0.001) and high VAT/SAT ratio (*Pc* < 0.001). Also, pCAD patients with the *UCP2 -866* (rs659366) *AA* genotype showed increased levels of VAF (*Pc* = 0.003), low levels of SAF (*Pc* = 0.001) and high VAT/SAT ratio (*Pc* = 0.002), whereas pCAD patients with the *UCP3 -55* (rs1800849) *TT* genotype showed high levels of VAF (*Pc* = 0.002).

**Table 4:** Association of the *UCP* polymorphisms with subcutaneous, visceral and total fat.

	*UCP2 55 TT*			*UCP2 -866 AA*			*UCP3 -55 TT*		
	pCAD	Control	*Pc*	pCAD	Control	*Pc*	pCAD	Control	*Pc*
VAT/SAT	0.682 (0.14-2.25)	0.547 (0.15-2.54)	**< 0.001**	0.703 (0.14-2.11)	0.545 (0.14-3.21)	**0.002**	0.696 (0.14-4.52)	0.561 (0.14-3.21)	0.247
SAF	263.51 (28-639)	310.14 (66-663)	**0.001**	255.07 (78-722)	305.12 (66-674)	**0.001**	260.92 (58-774)	307.35 (64-713)	0.524
VAF	172.66 (35-445)	149.80 (41-464)	**0.002**	175.49 (43-407)	144.64 (40-473)	**0.003**	172.43 (23-504)	150.05 (27-473)	**0.002**
TAF	436.17 (143-956)	459.81 (145-958)	0.622	430.58 (143-827)	449.74 (134-1032)	0.571	432.84 (113-1031)	457.49 (91-1032)	0.744

VAT/SAT: ratio visceral to subcutaneous adipose tissue ratio. SAF: Subcutaneous Abdominal Fat, VAF: Visceral Abdominal Fat, TAF: Total Abdominal Fat.

Bonferroni correction was made multiplying by 4 comparisons (*Pc*). Significant p values are in bold.

The association of the *UCP* polymorphisms with cardiovascular risk factors and metabolic parameters was analyzed (Table 5). The *UCP2 A55V* (rs660339) polymorphism was associated with BMI > 30 kg/cm^2^ (OR = 1.64, 95% CI: 1.30-2.06, *Pc*_*rec*_ = 0.003; OR = 1.44, 95% CI: 1.15-1.80, *Pc*_*add*_ = 0.001) and LDL-C > 130 mg/dL (OR = 3.01, 95% CI: 2.40-3.78, Pc_add_ = 0.015). The *UCP2 -866* (rs659366) was associated with BMI > 30 kg/cm^2^ (OR = 1.45, 95% CI: 1.16-1.81, *Pc*_*add*_ = 0.030), blood pressure > 140 mmHg (OR = 1.72, 95% CI: 1.23-2.41, *Pc*_*add*_ = 0.049), hypercholesterolemia (OR = 3.41, 95% CI: 2.22-5.26, *Pc*_*rec*_ = 0.006; OR = 1.65, 95% CI: 1.26-2.17, *Pc*_*add*_ = 0.038), and T2DM (OR = 2.72, 95% CI: 1.64-4.50, *Pc*_*rec*_ = 0.001; OR = 2.85, 95% CI: 1.74-4.65, *Pc*_*add*_ < 0.001). Finally, the *UCP3 -55 C/T* (rs1800849) polymorphism was associated with blood pressure > 140 mmHg (OR = 6.53, 95% CI: 5.16-8.27, *Pc*_*rec*_ = 0.007; OR = 6.55, 95% CI: 5.21-8.31, *Pc*_*add*_ = 0.011), hypercholesterolemia (OR = 2.53, 95% CI: 1.92-3.44, *Pc*_*rec*_ = 0.007; OR = 2.56, 95% CI: 1.95-3.49, *Pc*_*add*_= 0.011), hypertriglyceridemia (OR = 1.99, 95% CI: 1.61-2.53, *Pc*_*rec*_ = 0.006; OR = 2.01, 95% CI: 1.69-2.62, *Pc*_*add*_ = 0.010), and T2DM (OR = 2.26, 95% CI: 1.70-3.01, *Pc*_*rec*_ = 0.007; OR = 2.30, 95% CI: 1.74-3.06, *Pc*_*add*_ = 0.011).

**Table 5:** Association of *UCP* polymorphisms with cardiovascular risk factors.

		*UCP2 A55V*			*UCP2 -866*			*UCP3 -55C/T*		
	Model	*Pc*	OR	95%CI	*Pc*	OR	95%CI	*Pc*	OR	95%CI
BMI >30 kg/cm^2^	Recessive	0.003	1.64	1.30-2.06	0.063	1.87	1.18-2.45	0.540	2.50	0.42-12.76
	Additive	**0.001**	1.44	1.15-1.80	**0.030**	1.45	1.16-1.81	0.197	1.60	0.88-2.09
HT >140 mmHg	Recessive	0.069	1.84	0.99-3.42	0.221	2.03	1.05-3.93	**0.007**	6.53	5.16-8.27
	Additive	0.052	1.33	1.00-1.77	**0.049**	1.72	1.23-2.41	**0.011**	6.55	5.21-8.31
Hypercholesterolemia > 160 mg/dL	Recessive	0.051	0.23	0.15-0.36	**0.006**	3.41	2.22-5.26	**0.007**	2.53	1.92-3.44
	Additive	0.062	0.22	0.18-0.28	**0.038**	1.65	1.26-2.17	**0.011**	2.56	1.95-3.49
LDL-C >130 mg/dL	Recessive	0.070	0.23	0.14-0.37	0.096	1.96	1.22-3.16	0.618	2.00	0.42-9.51
	Additive	**0.015**	3.01	2.40-3.78	**0.030**	0.14	0.10-0.19	0.236	3.03	1.94-4.63
Hypertriglyceridemia >150 mg/dL	Recessive	0.066	0.57	0.39-0.84	0.151	2.63	1.76-3.92	**0.006**	1.99	1.61-2.53
	Additive	0.059	0.53	0.44-0.65	0.100	2.20	1.81-2.67	**0.010**	2.01	1.69-2.62
Hypoalphalipoproteinemia < 40 mg/dL	Recessive	0.168	1.56	1.07-2.26	0.910	1.04	0.71-1.52	0.862	1.37	0.38-4.86
	Additive	0.054	1.33	1.11-1.60	0.296	0.90	0.75-1.08	0.984	1.01	0.72-1.42
T2DM	Recessive	0.086	2.08	1.22-3.52	**0.001**	2.72	1.64-4.50	**0.007**	2.26	1.70-3.01
	Additive	0.050	2.09	1.63-2.69	**< 0.001**	2.85	1.74-4.65	**0.011**	2.30	1.74-3.06

All associations were tested using logistic regression adjusting for age, gender, smoking habits and physical activity. BMI: body mass index, HT: hypertension; LDL-C: Low density lipoprotein-cholesterol; T2DM: Type 2 diabetes mellitus. Bonferroni correction was made multiplying by 7 comparisons (Pc). Significant p values are in bold.

The analysis of haplotypes showed one block composed of two polymorphisms in linkage disequilibrium (D’=0.964, r^2^= 0.881), *UCP2 A55V* (rs660339) and *UCP2 -866* (rs659366). The analyses showed three different possible allele combinations (*GC*, *AT* and *AC*). Nonetheless, the distribution of these haplotypes was similar in both pCAD patients and healthy controls (data no shown).

## Discussion

It is known that many molecular mechanisms are implicated in cardiovascular damage. These mechanisms include advanced glycation end products (AGE), protein kinase C, polyol and hexosamine pathway. They appear to be associated with an overproduction of superoxide by the mitochondrial electron transport chain (Gioli-Pereira *et al.*, 2013). In this phenomenon participate the uncoupling proteins (UCPs) and in consequence, the genes that encode these molecules (*UCP1*, *UCP2* and *UCP3*) are regarded as candidate genes for obesity, T2DM and cardiovascular disease. Therefore, the identification of variants present in *UCP* genes and their relation to visceral and subcutaneous adipose tissue and lipid parameters in patients with CAD can assist in evaluating their participation in predisposition to this disease. Previous studies have explored the effects of these genes on various traits in different populations (Holdys *et al.*, 2013). However, results are inconsistent concerning the association with dyslipidemic parameters.

In the present study, we determined whether the *UCP2 Ala55Val* (rs660339), *UCP2* -866G/A (rs659366), and *UCP3 -55C/T* (rs1800849) polymorphisms are associated with risk of developing pCAD or clinical/metabolic parameters. We found a higher frequency in the minor allele in the *UCP2 -866* (rs659366) variants in the pCAD group, with a significant difference in the recessive model. Similar allele and genotype frequencies have been found in Asian populations (Oktavianthi *et al.*, 2012). In spite that our study included an important number of patients and healthy controls, the statistical power for two polymorphisms was low [20% for *UCP2 Ala55Val* (*C/T* rs660339 and 6% for *UCP3* -*55C/T* (rs1800849)]. Thus, the non significant results with these polymorphisms could be due to this lack of statistical power.

Other studies have explored the association of *UCP* genes with abdominal obesity and lipid levels in several populations; yet, results have been inconclusive. In addition, most of the studies have been based on indices of abdominal obesity like waist circumference, waist-to-hip ratio (WHR) and body mass index. In our study, we ascertain the abdominal and visceral fat by computed tomography. Although no BMI difference between our groups was found, the analysis showed association between the *UCP* variants and SAF and VAF levels. These results indicate an increase in the VAF in pCAD subjects depending of the *UCP2 55* (rs660339) *TT*, *UCP2 -866* (rs659366) *AA* and *UCP3 -55TT* (rs1800849) genotypes. Some studies have reported that a high fat diet increases *UCP2* mRNA expression in white adipose tissue in some mouse strains (Surwit *et al.*, 1998). In Caucasoid and Indian populations, the *UCP3 -55* (rs1800849) *T* allele was associated with abdominal obesity (Cassel *et al.*, 2000; Herrmann *et al.*, 2003), meanwhile in Asian population the *C* allele present in the *CGTACC* haplotype was associated with this condition (Cha *et al.*, 2007). Also, the *UCP2 -866* (rs659366) *A* allele was associated with lower abdominal obesity indices in Caucasian population (Salopuro *et al.*, 2009). Despite the consistency of these findings with previous reports, contradictory results have been reported in different populations (Pedersen *et al.*, 2005; Qin *et al.*, 2013; Xu, *et al.*, 2011). This could be due to diet and life style, genetic charge between different ethnic groups, and also to the sample size, source of controls and genotyping methods.

Previous studies have found that *UCP2 A55V* (rs660339) polymorphism is associated with cardiovascular event risk in patients with CAD and dysglycemia (Gioli-Pereira *et al.*, 2013). Alternatively, other research groups have reported that subjects carrying the *Val/Val* genotype in this polymorphism appear to have the following traits: a lower degree of uncoupling of the mitochondrial internal membrane, lower energy expenditure (Astrup *et al.*, 1999), higher exercise energy efficiency (Buemann *et al.*, 2001), higher metabolic rate, high atherogenic index, increased susceptibility to obesity and T2DM and greater weight loss than subjects with the Ala allele (De Souza *et al.*, 2013; Brondani *et al.*, 2014). In our study, the *UCP2 A55V* (rs660339) polymorphism was associated with BMI > 30 Kg/cm^2^.

Also, Pedersen *et al.* (2005) reported that *UCP2* and *UCP3* increase the serum lipid levels and abdominal obesity index, and so contribute to T2DM. Hence, different studies have described conflicting results: some studies found a negative association between *UCP* genes and obesity, while others found a positive association or none at all (Astrup *et al.*, 1999; Esterbauer *et al.*, 2001; Xu *et al.*, 2011; Qin *et al.*, 2013). The *UCP2 -866GA* (rs659366) polymorphism has been associated with prevalence of obesity (Pedersen *et al.*, 2005), and decreased or increased risk of T2DM (Bulotta *et al.*, 2005; Cheurfa *et al.*, 2008). In our study, this polymorphism was associated with high BMI and increased risk of T2DM.

In a meta-analysis, Qin *et al.* (2013) documented that *UCP2 A55V* (rs660339) and *UCP3 -55C/T* (rs1800849) polymorphisms were associated with T2DM susceptibility in Asian populations, whereas *UCP2 -866G/A* (rs659366) was linked to obesity in the European, but not the Asian population. Further, the *UCP2 -55 T* (rs660339) allele has been associated with greater energy expenditure, T2DM and cardiovascular risk, the *UCP2 -866 A* (rs659366) allele has been associated with obesity and increased cardiovascular risk and T2DM in obese individuals, meanwhile, the *UCP3 T* (rs1800849) allele was linked to BMI, and reduced energy expenditure (Buemann *et al.*, 2001; Reis *et al.*, 2004; Gable *et al.*, 2006).

In the present study, the *UCP2* and *UCP3* variants were associated with an increase in visceral fat and an increased risk of pCAD. A possible explanation is that *UCP* variants attenuate mRNA levels which leads to a diminished fat oxidation, consequently, this augments the risk of pCAD. Further investigation is merited to assess the mRNA levels and the oxidation patients with pCAD in the context of *UCP2* and *UCP3* variants.

Many studies have established that the effect of the genetic polymorphisms on *UCP2* and *UCP3* vary depending on physical activity and lifestyle (Berentzen *et al.*, 2005; Holdys *et al.*, 2013; Gronek *et al.*, 2013). Specifically, in the present study, the analyses were adjusted for confounders, such as gender, age, smoking habit, and physical activity.

In conclusion, our study revealed there is a direct relationship between visceral and abdominal fat accumulation depending on *UCP* polymorphisms, which seem to be related to BMI, high levels of cholesterol, triglycerides and T2DM in pCAD patients.
